# Fine mapping of *qHd1*, a minor heading date QTL with pleiotropism for yield traits in rice (*Oryza sativa* L.)

**DOI:** 10.1007/s00122-014-2395-7

**Published:** 2014-09-16

**Authors:** Jun-Yu Chen, Liang Guo, Huan Ma, Yu-Yu Chen, Hong-Wei Zhang, Jie-Zheng Ying, Jie-Yun Zhuang

**Affiliations:** State Key Laboratory of Rice Biology and Chinese National Center for Rice Improvement, China National Rice Research Institute, Hangzhou, 310006 China

## Abstract

*****Key message***:**

**A minor QTL for heading date located on the long arm of rice chromosome 1 was delimitated to a 95.0-kb region using near isogenic lines with sequential segregating regions.**

**Abstract:**

Heading date and grain yield are two key factors determining the commercial potential of a rice variety. In this study, rice populations with sequential segregating regions were developed and used for mapping a minor QTL for heading date, *qHd1*. A total of 18 populations in six advanced generations through BC_2_F_6_ to BC_2_F_11_ were derived from a single BC_2_F_3_ plant of the *indica* rice cross Zhenshan 97 (ZS97)///ZS97//ZS97/Milyang 46. The QTL was delimitated to a 95.0-kb region flanked by RM12102 and RM12108 in the terminal region of the long arm of chromosome 1. Results also showed that *qHd1* was not involved in the photoperiodic response, having an additive effect ranging from 2.4 d to 2.9 d observed in near isogenic lines grown in the paddy field and under the controlled conditions of either short day or long day. The QTL had pleiotropic effects on yield traits, with the ZS97 allele delaying heading and increasing the number of spikelets per panicle, the number of grains per panicle and grain yield per plant. The candidate region contains ten annotated genes including two genes with functional information related to the control of heading date. These results lay a foundation for the cloning of *qHd1*. In addition, this kind of minor QTLs could be of great significance in rice breeding for allowing minor adjustment of heading date and yield traits.

**Electronic supplementary material:**

The online version of this article (doi:10.1007/s00122-014-2395-7) contains supplementary material, which is available to authorized users.

## Introduction

In rice, heading date is a crucial determinant for adaption to different cultivation area and cropping seasons, and grain yield is an immediate indicator of the productivity. They are key factors determining the commercial potential of a rice variety. Mapping of quantitative trait loci (QTLs) for heading date, grain yield and yield component traits in rice has resulted in remarkable progresses on the genetic basis underlying the natural variation of these traits. A total of 711 QTLs for heading date and 2060 QTLs for yield traits have been documented in the Gramene database (http://archive.gramene.org/qtl/; Monaco et al. 2014). Nevertheless, only a small proportion of these QTLs have been fine mapped or cloned (Bai et al. 2012; Guo et al. 2013b).

Generally, genetic loci chosen for fine mapping and cloning have been those considered major QTLs due to large effects observed across different genetic backgrounds and environments. Many QTLs considered minor for having smaller individual effect which are often inconsistent across environments, thus remain poorly characterized. However, major QTLs for heading date and grain yield have shown a common association between delayed heading and increased grain yield, such as *Ghd7* (Xue et al. 2008; Weng et al. 2014), *DTH8/Ghd8/qHY*-*8*/*LH8* (Wei et al. 2010; Yan et al. 2011; Cai et al. 2012; Chen et al. 2014)*, Hd1* (Zhang et al. 2012) and *Ghd7.1* (Yan et al. 2013). The utilization of these QTLs would significantly influence the regional and seasonal adaption of a rice variety.

On the other hand, minor QTLs could be of great significance in rice breeding for allowing minor adjustment or fine tuning of the traits. In this regard, minor QTLs that slightly delay or promote heading can allow plants to sometimes flower later, making full use of the temperature and sunlight of longer growing seasons, but other times allow earlier heading times as needed to avoid abiotic stress from high or low temperature during flowering or grain filling. For example, the study of *DTH2* has revealed the great value of such a minor QTL in the historical expansion of rice into northern production areas of Asia noted for long-day growing seasons (Wu et al. 2013). In addition, slow increase of grain yield in the past few decades has been witnessed for rice varieties released in China (Yang et al. 2010; Yu et al. 2012), suggesting that the pyramiding of minor QTLs might be a main approach to increase the yield potential before a new breakthrough on the germplasm exploitation and gene deployment turns up.

We herein report the detection and fine mapping of a minor QTL for heading date of rice, *qHd1*, which was located in the terminal region of the long arm of chromosome 1. This QTL was not detected in primary QTL mapping using recombinant inbred lines of an *indica* rice cross between maintainer line Zhenshan 97 (ZS97) and restorer line Milyang 46 (MY46) (Zhang et al. 2011), but its effect was observed in two BC_2_F_6_ populations of the cross ZS97^3^/MY46 and validated in populations with higher homogenous background.

## Materials and methods

### Plant materials

**Fig. 1:**
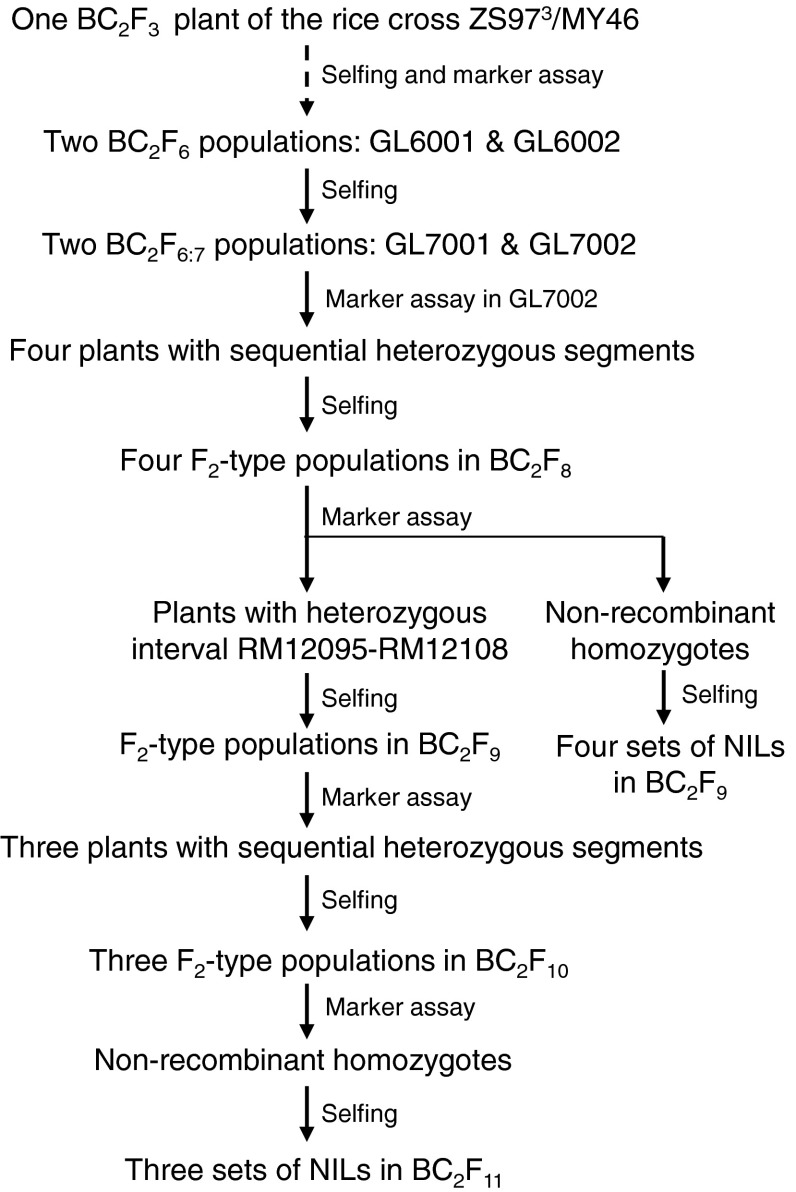
Development of the rice materials used in this study

A total of 18 segregating populations were used, including 17 grown in the paddy field (Table 1) and one tested in the phytotron. They were constructed from the rice cross ZS97^3^/MY46 as described below and illustrated in Fig. 1.

**Table 1:** Rice populations and field experiments

Generation	Name	Segregating region	Sample^a^	Location and growing season^b^	Trait measured^c^
BC_2_F_6_	GL6001	RM12026–RM12285	161 plants	LS: Dec 2010–Apr 2011	HD
BC_2_F_6_	GL6002	RM12026–RM12108	222 plants	LS: Dec 2010–Apr 2011	HD
BC_2_F_6:7_	GL7001	RM12026–RM12285	161 lines	HZ: May–Sep 2011	HD
BC_2_F_6:7_	GL7002	RM12026–RM12108	222 lines	HZ: May–Sep 2011	HD
BC_2_F_8_	C8001	RM12026–RM12072	250 plants	LS: Dec 2011–Apr 2012	HD
BC_2_F_8_	C8002	RM12055–RM12108	250 plants	LS: Dec 2011–Apr 2012	HD
BC_2_F_8_	C8003	RM12095–RM12108	246 plants	LS: Dec 2011–Apr 2012	HD
BC_2_F_8_	C8004	RM12095–RM12108	244 plants	LS: Dec 2011–Apr 2012	HD
BC_2_F_9_	C1	RM12026–RM12072	29 lines of ZS97, 31 lines of MY46	HZ: May–Sep 2012	HD
BC_2_F_9_	C2	RM12055–RM12108	28 lines of ZS97, 32 lines of MY46	HZ: May–Sep 2012	HD
BC_2_F_9_	C3	RM12095–RM12108	28 lines of ZS97, 36 lines of MY46	HZ: May–Sep 2012	HD, yield traits
BC_2_F_10_	CJ101	RM12095–Wn40348	297 plants	LS: Dec 2012–Apr 2013	HD
BC_2_F_10_	CJ102	Wn40348–RM12108	298 plants	LS: Dec 2012–Apr 2013	HD
BC_2_F_10_	CJ103	RM12108	300 plants	LS: Dec 2012–Apr 2013	HD
BC_2_F_11_	CJ1	RM12195–Wn40348	50 lines of ZS97, 50 lines of MY46	HZ: May–Sep 2013 HD, yield traits	
BC_2_F_11_	CJ2	Wn40348–RM12108	50 lines of ZS97, 50 lines of MY46	HZ: May–Sep 2013 HD, yield traits	
BC_2_F_11_	CJ3	RM12108	50 lines of ZS97, 50 lines of MY46	HZ: May–Sep 2013 HD, yield traits	

^a^ZS97, Zhenshan 97 homozygote; MY46, Milyang 46 homozygote

^b^LS, Lingshui, Hainan province; HZ, Hangzhou, Zhejiang province

^c^HD, heading date (d); the yield traits measured were number of spikelets per panicle (NSP), number of grains per panicle (NGP), 1,000-grain weight (TGW, g) and grain yield per plant (GY, g)

**Fig. 2:**
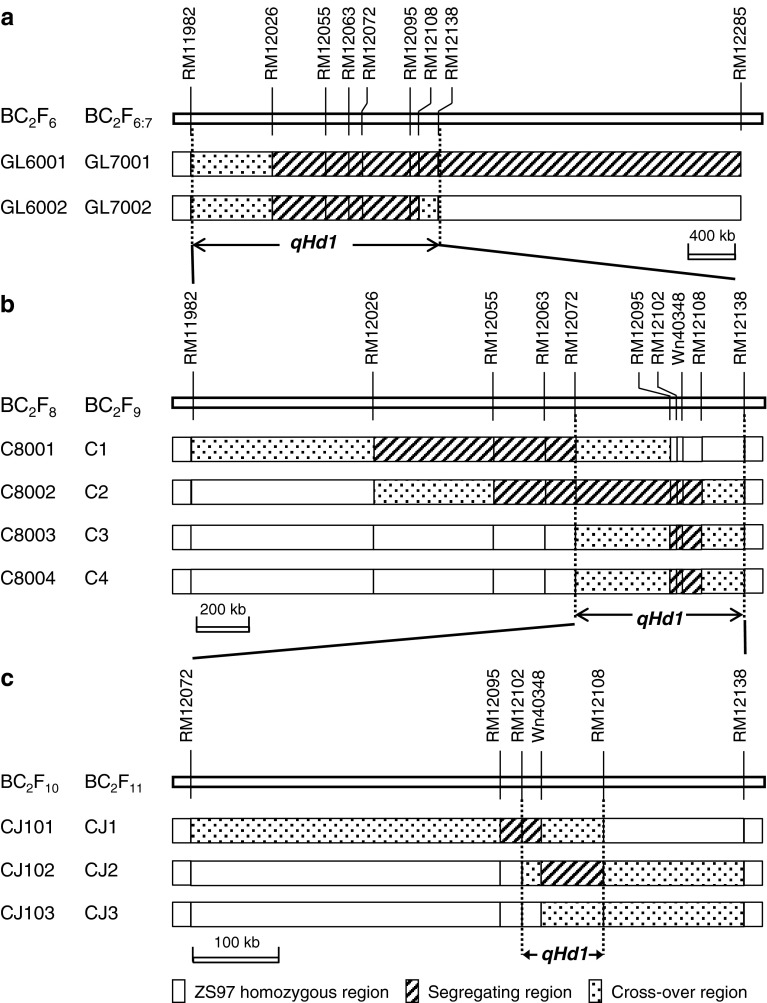
Segregating populations used for mapping *qHd1*, showing sequential segregating regions in each generation and the delimitation of *qHd1* to a 95.0-kb region flanked by RM12012 and RM12108. **a** Two sets of BC_2_F_6_ and BC_2_F_6:7_ populations; **b** four F_2_-type populations in BC_2_F_8_ and four sets of near isogenic lines in BC_2_F_9_; **c** three F_2_-type populations in BC_2_F_10_ and three sets of near isogenic lines in BC_2_F_11_

In a previous study (Guo et al. 2013a), three sets of populations were derived from a single BC_2_F_3_ plant of ZS97^3^/MY46. Among them, two BC_2_F_6_ populations were selected as the starting materials for the present study. They were segregated in the intervals RM12026–RM12285 and RM12026–RM12108, respectively (Fig. 2a). The two BC_2_F_6_ populations, as well as their BC_2_F_6:7_ offspring, were used for QTL analysis on heading date. Henceforth, results of QTL detection were followed in each generation to develop new populations for validating and fine mapping *qHd1*.

In BC_2_F_7_, four plants with sequential heterozygous segments extending from RM12026 to RM12108 were identified, from which four BC_2_F_8_ populations were derived and named C8001, C8002, C8003 and C8004, respectively (Fig. 2b). They were assayed with DNA markers in the target interval. In each population, non-recombinant homozygotes were identified and selfed to produce homozygous lines. Four sets of near isogenic lines (NILs) in BC_2_F_9_ were established and named C1, C2, C3 and C4, respectively (Fig. 2b).

In the mean time, BC_2_F_8_ plants carrying a heterozygous segment extending from RM12095 to RM12108 were selected. The resultant BC_2_F_9_ populations were assayed with DNA markers in the target interval. Three plants with sequential heterozygous segments were identified, from which three BC_2_F_10_ populations were developed and named CJ101, CJ102 and CJ103, respectively (Fig. 2c). Then, non-recombinant homozygotes were identified and selfed to produce homozygous lines. Three sets of NILs in BC_2_F_11_ were developed and named CJ1, CJ2 and CJ3, which were segregated in regions covering RM12095–Wn40348, Wn40348–RM12108 and RM12108, respectively.

### Field experiments

The rice populations were tested at experimental fields of the China National Rice Research Institute located either in Hangzhou, Zhejiang, or Lingshui, Hainan (Table 1). In all the trials, the planting density was 16.7 cm between plants and 26.7 cm between rows.

Heading date (HD) was scored for each of the populations. For the two BC_2_F_6_, four BC_2_F_8_ and three BC_2_F_10_ populations, HD was measured on a single-plant basis. For the remaining populations, a randomized complete block design with two replications was applied. In each replication, one line was grown in a single row of 12 plants. HD was scored for each plant and averaged for each replication.

Four yield traits, including number of spikelets per panicle (NSP), number of grains per panicle (NGP), 1,000-grain weight (TGW) and grain yield per plant (GY), were also measured for one of the NIL sets in BC_2_F_9_ and all the three NIL sets in BC_2_F_11_. For NIL set C3 in BC_2_F_9_, five middle plants of each row were harvested in bulk and measured for the four yield traits. For the three NIL sets in BC_2_F_11_, two main panicles in each of the five middle plants were bulk-harvested and measured for NSP, NGP and TGW. The remaining panicles of the five middle plants were also harvested and added for the measurement of GY.

### Phytotron experiments

To determine the photoperiodic response of *qHd1*, NIL set C4 consisting of two homozygous genotypes differing in a 120.2 kb region flanked by RM12095 and RM12108 (Fig. 2b) was grown in controlled chambers, under short-day (SD, 10 h light/14 h dark, 12 h 28 °C/12 h 23 °C) and long-day (LD, 14 h light/10 h dark, 12 h 28 °C/12 h 23 °C) conditions, respectively. Eight lines for each genotype with eight plants per line were grown. HD was scored for each plant.

### DNA marker analysis

Total DNA was extracted following the method of Zheng et al. (1995). PCR amplification was performed according to Chen et al. (1997). The products of the SSR markers were visualized on 6 % non-denaturing polyacrylamide gels using silver staining, and that of the InDel marker were visualized on 2 % agarose gels using Gelred staining. All the SSR markers were selected from the Gramene database (http://www.gramene.org/). The InDel marker Wn40438 was designed according to the difference of the genomic sequences between ZS97 and MY46 detected by the whole-genome re-sequencing (Forward primer: ACATGTGTAGCATTAACAAC; Reverse primer: ATGATTTTGTTCAACCTTGG).

Parental survey of polymorphism was performed using SSR markers located in the terminal region of chromosome 1. Polymorphic markers were used to determine new segregating regions of the two BC_2_F_6_ populations, by testing two DNA bulks of 10 plants from each population. DNA markers shown in Fig. 2a were used for the selection of sequential heterozygotes in BC_2_F_7_, and those presented in Fig. 2b, c for constructing the NIL sets in BC_2_F_9_ and BC_2_F_11_, respectively.

In QTL mapping using the two BC_2_F_6_ populations and their BC_2_F_7_ progenies, RM12026 and RM12285 were used to test the 161 plants of GL6001, while RM12026 and RM12063 were chosen to assay the 222 plants of GL6002. In QTL mapping using the BC_2_F_8_ and BC_2_F_10_ populations, one marker was applied for each population. In BC_2_F_8_, the markers used were RM12026 for C8001, RM12063 for C8002, and RM12102 for C8003 and C8004. In BC_2_F_10_, the markers used were RM12102 for CJ101, and RM12108 for CJ102 and CJ103.

### Data analysis

Mapmaker/Exp 3.0 (Lander et al. 1987) was used for the map construction of the two BC_2_F_6_ populations, and the genetic distances in centiMorgan (cM) were derived by Kosambi function. QTL analysis was performed with Windows QTL Cartographer 2.5 (Wang et al. 2012), in which the interval mapping was used for the BC_2_F_6_ and BC_2_F_6:7_ populations, while the single-marker analysis was used for the BC_2_F_8_ and BC_2_F_10_ populations. Using 1,000 permutation tests, the critical LOD values at *P* = 0.01 were determined, ranging from 1.9 to 4.1.

Two-way ANOVA was performed for the six NIL sets in BC_2_F_9_ or BC_2_F_11_ which were planted in the paddy field in Hangzhou (Table 1). Phenotypic differences between the two homozygous genotypic groups in each NIL set were tested using SAS procedure GLM (SAS Institute Inc 1999) as described previously (Dai et al. 2008). When significant differences were detected (*P* < 0.01), the same model was applied to estimate the genetic effect of the QTL, including additive effect and the proportion of phenotypic variance explained.

For NIL set C4 which was grown in the controlled chamber, one-way ANOVA was conducted to test the phenotypic differences between the two homozygous genotypic groups under SD and LD conditions, respectively.

## Results

### Detection of *qHd1* using BC_2_F_6_ and BC_2_F_6:7_ populations

The two BC_2_F_6_ populations were previously found to be segregated in the intervals RM11448–RM11615 and RM11448–RM11787, and meanwhile to be ZS97 homozygous in the subsequent regions extended to RM11982, respectively (Guo et al. 2013a). An additional region towards the terminal end of the long arm of chromosome 1 was determined in the present study, extending from RM12026 that is 696 kb apart from RM11982, to RM12285 having a distance of 106 kb from the terminal end.

The regions RM12026–RM12285 and RM12026–RM12108 were found to be segregated in GL6001 and GL6002, respectively (Fig. 2a), and the segregation is highly distorted. No heterozygotes were found in GL6001, with the genotype ratio of ZS97:MY46 appearing to be 56:105 at RM12026 and 32:129 at RM12285. Three genotypes were detected in GL6002, with the genotype ratio of ZS97:MY46:heterozygote appeared to be 72:102:48 at RM12026, and 73:102:47 at RM12063.

**Fig. 3:**
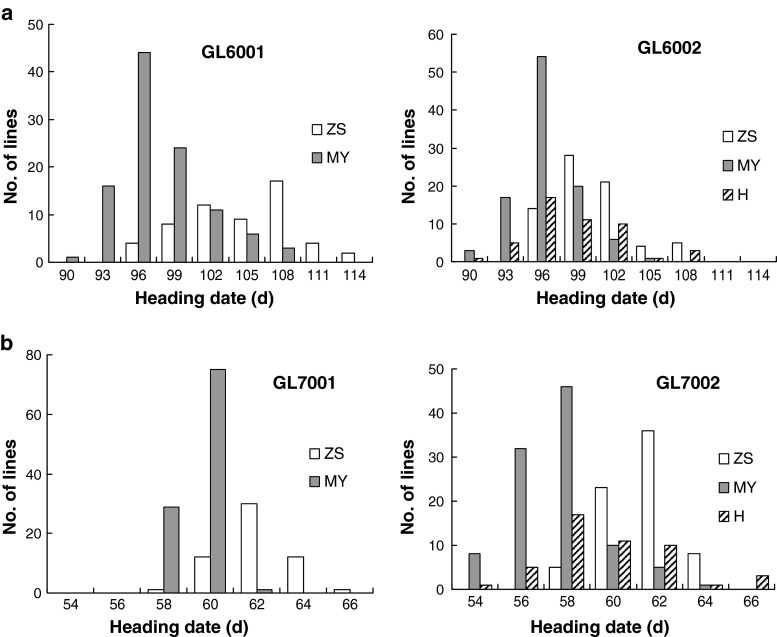
Frequency distribution of heading date in the two sets of BC_2_F_6_ and BC_2_F_7_ populations. ZS, MY and H represent the homozygote for Zhenshan 97, homozygote for Milyang 46 and the heterozygote as defined by the genotype at RM12026, respectively

Heading date was continuously distributed in the BC_2_F_6_ and BC_2_F_6:7_ populations, but it exhibited a single-QTL segregation when each population was classified based on the genotype of the common segregating marker RM12026 (Fig. 3). In the GL6001 population, the MY46 homozygous plants tended to flower earlier than the ZS97 homozygous plants, and the separation between the two groups was much more obvious in GL7001 when the phenotype was measured in the replicated trial. In GL6002 and GL7002, the MY46 homozygotes also tended to flower earlier than the ZS97 homozygotes, while the heterozygotes were more evenly distributed and scattered in the whole range.

Two segmental linkage maps were constructed for GL6001 and GL6002, spanning 15.4 cM for the interval RM12026–RM12285 and 1.1 cM for the interval RM12026–RM12063, respectively. They were used to detect the QTL responsible for the segregation of heading date in the two sets of BC_2_F_6_ and BC_2_F_6:7_ populations. A QTL was detected in each population, with the MY46 allele always promoting flowering (Table 2). The phenotypic variance explained was 49.5 and 25.0 % in the BC_2_F_6_ populations GL6001 and GL6002, and 68.3 and 48.3 % in the BC_2_F_6:7_ populations GL7001 and GL7002, respectively, with the additive effect ranging from 1.2 to 3.0 d. This putative QTL was named *qHd1*. In addition to the common segregating region RM12026–RM12108, the two flanking cross-over regions RM11982–RM12026 and RM12108–RM12138 may also segregated due to their unknown genotypes, thus *qHd1* was located at RM11982–RM12138 (Fig. 2a).

**Table 2:** QTL analysis for heading date in populations of four different generations

Generation	Name	Interval/Maker analyzed	Location	*LOD*	*A* ^a^	*D* ^b^	D/[A]^c^	*R* ^2^(%)^d^
BC_2_F_6_	GL6001	RM12026–RM12285	Lingshui	20.07	−3.0	n.a	n.a	49.5
BC_2_F_6:7_	GL7001	RM12026–RM12285	Hangzhou	35.91	−1.2	n.a	n.a	68.3
BC_2_F_6_	GL6002	RM12026–RM12063	Lingshui	13.83	−2.0	0.1	0.05	25.0
BC_2_F_6:7_	GL7002	RM12026–RM12063	Hangzhou	31.96	−1.9	−0.2	−0.11	48.3
BC_2_F_8_	C8001	RM12026	Lingshui	0.10				
BC_2_F_8_	C8002	RM12063	Lingshui	12.57	−1.2	0.3	0.25	20.7
BC_2_F_8_	C8003	RM12102	Lingshui	6.22	−1.3	−0.1	−0.08	11.0
BC_2_F_8_	C8004	RM12102	Lingshui	14.99	−1.7	0.4	0.24	24.6
BC_2_F_10_	CJ101	RM12102	Lingshui	21.52	−2.9	−0.2	−0.07	28.4
BC_2_F_10_	CJ102	RM12108	Lingshui	23.65	−2.9	−0.3	−0.10	31.2
BC_2_F_10_	CJ103	RM12108	Lingshui	0.07				

^a^Additive effect of replacing a Zhenshan 97 allele by a Milyang 46 allele

^b^Dominance effect; *n.a.* not available

^c^Degree of dominance; *n.a.* not available

^d^Proportion of phenotypic variance explained by the QTL effect

### Validation of *qHd1*

The effect of *qHd1* on heading date was validated in BC_2_F_8_ and BC_2_F_10_ populations which were segregated in the expected 1:2:1 ratio. Results of the *χ* ^*2*^ test and the average heading date of the three genotypic groups in each population are presented in Table S1.

In BC_2_F_8_, significant association between marker genotypes and heading date was detected in all the population except C8001 (Table 2), suggesting that *qHd1* was located in the common segregating region of C8002, C8003 and C8004. This QTL explained 11.0–24.6 % of the phenotypic variance in the three populations, with the MY46 allele promoting heading by 1.2–1.7 d and the dominance effect ranging from −0.1 d to 0.4 d. These effects were consistent with those detected in the BC_2_F_6_ and BC_2_F_6:7_ populations. Similar results were obtained using the three BC_2_F_10_ populations. The effects of *qHd1* were again highly consistent with those detected in previous generations (Table 2). It could be concluded that *qHd1* is a minor QTL with the additive genetic action mode.

### Fine mapping of *qHd1*

The six NIL sets tested in the replicated trials in Hangzhou in 2012 or 2013 (Table 1) were used to measure the effect of *qHd1* with lower random error and fine map *qHD1*. Results of two-way ANOVA for the heading date difference between two homozygous genotypic groups in each of the NIL sets are shown in Table 3.

**Table 3:** QTL analysis for heading date and yield traits using near isogenic lines

Generation	Name	Trait^a^	Phenotypic mean^b^		*P*	*A* ^c^	*R* ^2^ (%)^d^
			NIL^ZS97^	NIL^MY46^			
BC_2_F_9_	C1	HD	66.4	66.2	0.4852		
BC_2_F_9_	C2	HD	65.2	60.0	<0.0001	−2.6	77.4
BC_2_F_9_	C3	HD	65.9	61.0	<0.0001	−2.4	79.1
		NGP	114.1	106.2	<0.0001	−4.0	12.9
		NSP	141.1	130.6	<0.0001	−5.2	18.0
		TGW	25.4	25.3	0.5176		
		GY	22.2	19.7	<0.0001	−1.2	19.7
BC_2_F_11_	CJ1	HD	69.1	63.5	<0.0001	−2.8	74.2
		NGP	103.4	88.1	<0.0001	−7.7	20.6
		NSP	132.6	114.1	<0.0001	−9.2	22.3
		TGW	23.3	23.2	0.9133		
		GY	24.0	21.7	<0.0001	−1.1	10.0
BC_2_F_11_	CJ2	HD	69.1	64.0	<0.0001	−2.5	69.8
		NGP	110.1	93.6	<0.0001	−8.2	36.4
		NSP	139.1	121.6	<0.0001	−8.8	41.5
		TGW	22.6	23.3	0.3015		
		GY	23.9	21.8	<0.0001	−1.0	10.2
BC_2_F_11_	CJ3	HD	69.6	69.8	0.5986		
		NGP	108.5	108.8	0.8894		
		NSP	136.3	135.8	0.8421		
		TGW	22.7	22.2	0.1313		
		GY	22.0	24.5	0.1921		

^a^HD, Heading date (d); NSP, number of spikelets per panicle; NGP, number of grains per panicle; TGW, 1,000-grain weight (g); GY, grain yield per plant (g)

^b^NIL^ZS97^ and NIL^MY46^ are near isogenic lines with Zhenshan 97 and Milyang 46 homozygous genotypes in the segregating region, respectively

^c^Additive effect of replacing a Zhenshan 97 allele by a Milyang 46 allele

^d^Proportion of phenotypic variance explained by the QTL effect

Among the three NIL sets in BC_2_F_9_, significant variations on heading date were observed in C2 and C3 but not in C1, indicating that the allelic difference at *qHd1* between ZS97 and MY46 was present in C2 and C3 but absent in C1. Therefore, *qHd1* could be delimited to the region within the interval RM12072–RM12138, which included the region RM12095–RM12108 segregated in C2 and C3 but homozygous in C1, as well as the two flanking cross-over regions RM12072–RM12095 and RM12108–RM12138 due to their unknown genotypes (Fig. 2b). As estimated from C2 and C3, *qHd1* explained 77.4 and 79.1 % of the phenotypic variance, with the MY46 allele reducing heading date by 2.6 d and 2.4 d, respectively (Table 3).

Among the three NIL sets in BC_2_F_11_ which were segregated within the candidate interval RM12072–RM12138, significant genotypic effects on heading date were detected in CJ1 and CJ2 but not in CJ3. Obviously, *qHd1* was located in a place between RM12102 and RM12108 since no parts of this region were homozygous in CJ1 and CJ2 (Fig. 2c). This interval corresponds to a 95.0-kb region in the Nipponbare genome (www.gramene.org). As estimated from CJ1 and CJ2, *qHd1* explained 74.2 and 69.8 % of the phenotypic variance, with the MY46 allele reducing heading date by 2.8 d and 2.5 d, respectively.

### Photoperiodic response of *qHd1*

To investigate whether the *qHd1* was involved in the photoperiodic response of rice, NIL set C4 which consisted of two homozygous genotypes differing in the interval RM12095-RM12108 covering *qHd1* was examined in the controlled chambers. Under both the SD (10 h light/14 h dark) and LD (14 h light/10 h dark) conditions, highly significant difference (*P* < 0.0001) was shown between the heading date of the two genotypic groups (Table 4). The MY46 allele promoting flowering in both the SD and LD conditions, with the additive effect estimated as 2.9 d and 2.4 d, respectively. These results suggest that the allelic variation at *qHd1* did not affect the photoperiodic sensitivity of rice.

**Table 4:** The effect of *qHd1* estimated in controlled environments

Condition	Heading date^a^		*P*	*A* ^b^
	NIL^ZS97^	NIL^MY46^		
Short day	76.9	71.1	<0.0001	−2.9
Long day	85.1	80.4	<0.0001	−2.4

^a^NIL^ZS97^ and NIL^MY46^ are near isogenic lines with Zhenshan 97 and Milyang 46 homozygous genotypes in the interval RM12095–RM12108 covering *qHd1*, respectively

^b^Additive effect of replacing a Zhenshan 97 allele by a Milyang 46 allele

### Effect of *qHd1* on yield traits

The effect of *qHd1* on yield traits was first tested using one of the three NIL sets in BC_2_F_9_, C3 which consisted of two homozygous genotypes differing in the interval RM12095-RM12108 covering *Hd1* (Fig. 2b), and then validated using the three NIL sets planted in BC_2_F_11_ (Table 1). Results of the two-way ANOVA on the four yield traits are presented in Table 3.

In the NIL set C3 which was planted in the paddy field in 2012, significant genotypic effect was observed for all the four yield traits analyzed except TGW, indicating that *qHd1* had pleiotropic effects on yield traits. A consistent allelic direction was observed, with the ZS97 allele increasing NGP by 4.0, NSP by 5.2, and GY by 1.2 g, explaining 12.9 % of the phenotypic variance for NGP, 18.0 % for NSP, and 19.7 % for GY.

In the three NIL sets in BC_2_F_11_ which were planted in the paddy field in 2013, significant variations on NGP, NSP and GY were found in CJ1 and CJ2 which were segregated for *qHd1*, and it was not detected in CJ3 which was not segregated for *qHd1*. As estimated from CJ1 and CJ2, the contribution to the phenotypic variance was 20.6 and 36.4 % for NGP, 22.3 and 41.5 % for NSP, 10.0 and 10.2 % for GY, with the ZS97 allele increasing NGP by 7.7 and 8.2, NSP by 9.2 and 8.8, and GY by 1.1 and 1.0 g, respectively. Thus, the pleiotropism of *qHd1* on yield traits was confirmed.

## Discussion

In QTL analysis for heading date and yield traits of rice, a great attention has been paid to characterize major QTLs, whereas minor QTLs are poorly investigated. In this study, a minor QTL for heading date in rice, *qHd1*, was delimited into a 95.0-kb region flanked by RM12102 and RM12108 on the long arm of chromosome 1. It has also been shown that *qHd1* did not respond to photoperiod and had pleiotropic effects on yield traits including grain number, spikelet number and grain yield. It is noteworthy that the minor effect of *qHd1* on heading date has been stably detected across different generations, years and locations. Its allelic direction remained unchanged in all the trials, and the additive effect ranged from 2.4 d to 2.9 d as estimated from NIL sets which were either in the generation of BC_2_F_9_ or BC_2_F_11_, tested in 2012 or 2013, and examined in the paddy field or controlled environments.

### Candidate genes for *qHd1*

According to the Rice Annotation Project database (http://rapdb.dna.affrc.go.jp/) (Sakai et al. 2013), ten genes in the target region for *qHd1* defined by RM12102 and RM12108 were predicted. Six genes of them contain functional domains, and the others are hypothetical genes/proteins.

Two of the six genes with functional information are related to the control of heading date. Os01g0922800 encodes a protein containing a conserved SRF-like MADS domain and corresponds to the cloned heading date gene *OsMADS51*. However, *OsMADS51* has shown a photoperiodic sensitivity and affects heading date in SD conditions only (Kim et al. 2007). This is obviously different from *qHd1* which was found to be photoperiodic insensitive, suggesting that *OsMADS51* is non-allelic to *qHd1*. The other gene, Os01g0922600, is a member of *SQUAMOSA Promoter*-*Binding Protein*-*Like* (*SPL*) gene family that is known to participate in the regulation of multiple plant developmental processes, such as phase transition and flowering (Huijser and Schmid 2011) and branch formation (Jiao et al. 2010; Miura et al. 2010). Thus, Os01g0922600 could be a potential candidate for *qHd1*.

The remaining four genes with functional information are Os01g0923200 encoding a AT.I.24-6-like protein, Os01g0923300 encoding a cystathionine beta-synthase, Os01g0923600 encoding an ankyrin domain-containing protein, and Os01g0923700 encoding a histidine kinase-type protein. More work is needed to examine whether these genes could be candidate for *qHd1*.

Sequence comparison between ZS97 and MY46 showed no polymorphism for the coding sequence in each of the ten annotated genes (data not shown), suggesting that the minor effect at *qHd1* might result from the difference of the expression levels between the ZS97 allele and MY46 allele. Sequencing analysis of the promoter regions and expression analysis for each of the ten genes are underway to determine the most potential candidate underlying *qHd1*.

### Application of sequential residual heterozygotes in QTL fine mapping

Determination of critical recombination breakpoints based on the genotypes of phenotypic extremes has been frequently applied to fine map major QTLs such as *Hd1*, *Ghd7* and *DTH8/Ghd8* (Yano et al. 2000; Xue et al. 2008; Wei et al. 2010; Yan et al. 2011), and this is greatly relied on the clear classification of the phenotypes. More generally, substitution mapping using multiple NILs with introgressions covering different portions of the target region was used for QTL fine mapping, e.g., *gw3.1*, *qSS7*, *qLTG*-*9*, *qSV*-*1* and *qSV*-*5c* (Li et al. 2004, 2013; Qiu et al. 2012; Xie et al. 2014). Identification of sufficient amount of introgression lines for each segment required a great effort on phenotyping and genotyping.

In our study, a new strategy of QTL fine mapping was employed. A single residual heterozygote identified was selfed to produce a population segregated in the target region. Plants carrying heterozygous segments in a sequential order were identified from the population, which could be named sequential residual heterozygotes (SeqRHs). Populations with sequential segregating regions were produced from the selfed seeds of the SeqRHs. NIL sets of which each consisted of two homozygous genotypes differing in the corresponding heterozygous regions were developed. Significant and insignificant phenotypic variation between the two genotypic groups would be an indication of existence and absence of QTL segregation in the target region, respectively.

Generally, a step-by-step approach could be applied in QTL fine mapping using SeqRHs. First, a segregating population is derived from one residual heterozygote that carried a relatively large heterozygous segment. This population is used for validating the QTL effect in an isogenic background, and in the same time for the identification of SeqRHs with smaller heterozygous segments. Then, new populations are produced to narrow down the QTL region, and to identify new SeqRHs covering the narrowed region if necessary. Eventually, a QTL might be delimited in a region containing one or a few candidate genes.

This approach has an advantage of greatly reducing the cost of QTL fine mapping. While large populations are needed to screen sufficient recombinants in substitution mapping, as few as one residual heterozygote is required for a target region. Another advantage of this approach is the easy development of NILs with sufficient sample size for reliable phenotyping. A considerable quantity of lines could be easily identified for each genotype, since generally a few hundred seeds would be produced from a single rice plant.

### Potential of *qHd1* in rice breeding

Association of longer heading date with large panicle has been frequently observed in the fine mapping and cloning of major QTL for heading date in rice (Xue et al. 2008; Wei et al. 2010; Yan et al. 2011, 2013; Cai et al. 2012; Zhang et al. 2012). It has also been reported that the duration of panicle differentiation was closely related to the panicle size (Huang et al. 2006). The pre-flowering development in rice includes three successive phases (Chang et al. 1969): the basic vegetative phase (BVP), the photoperiod-sensitive phase (PSP), and the panicle-differentiation phase (PDP). Since *qHd1* is not involved in the photoperiodic response, it might affect heading through its influence on BVP or PDP. Moreover, *qHd1* exhibits pleiotropism for spikelet number and grain number, providing another evidence for the involvement of this QTL in the panicle differentiation during PDP.

In terms of the breeding application, selection of the genotypes at *qHd1* would be helpful for fine tuning heading date, so as to make a full use of the temperature and sunlight while avoiding abiotic stresses. In this field, the two SSR markers flanking *qHd1*, RM12102 and RM12108, and the InDel marker Wn40348 located between them, could be used to increase the breeding efficiency by marker-assisted selection.

#### **Author contributions**

JYZ designed the experiments and selected the rice materials. JYC and LG performed most of the experiments. HM and JZY developed new DNA markers. YYC and HWZ performed some of the phenotyping. JYC and JYZ analyzed the data and wrote the paper.

## Electronic supplementary material

Below is the link to the electronic supplementary material.

Supplementary material 1 (Xls 27 kb)
